# Supplementary material for: PEDOT Films Doped with Titanyl Oxalate as Chemiresistive and Colorimetric Dual-Mode Sensors for the Detection of Hydrogen Peroxide Vapor
Source: Sensors (Basel). 2023 Mar 14;23(6):3120. doi: 10.3390/s23063120 (PMC10051208; doi:10.3390/s23063120)
Supplement: Supplementary file 1 [file sensors-23-03120-s001.zip › sensors-2271538-supplementary.pdf]

Supporting Information

PEDOT Films Doped with Titanyl Oxalate as Chemiresistive and Colorimetric Dual-Mode Sensors for the Detection of Hydrogen Peroxide Vapor

Xiaowen Xie [1], Nan Gao [2], Matthew Hunter [3], Ling Zhu [1], Xiaomei Yang [4], Shuai Chen [1,2,*] and Ling Zang [3,4,*]

[1] Jiangxi Key Laboratory of Flexible Electronics and School of Pharmacy, Jiangxi Science & Technology Normal University, Nanchang 330013, China
[2] Jiangxi Engineering Laboratory of Waterborne Coating, Nanchang 330013, China
[3] Department of Materials Science and Engineering, University of Utah, Salt Lake City, UT 84112, USA
[4] Nano Institute of Utah, University of Utah, Salt Lake City, UT 84112, USA
* Correspondence: shuaichen@jxstnu.edu.cn (S.C.); lzang@eng.utah.edu (L.Z.); Tel.: +86-791-83832345 (S.C.); +1-801-587-1551 (L.Z.); Fax.: +1-801-581-4816 (L.Z.)

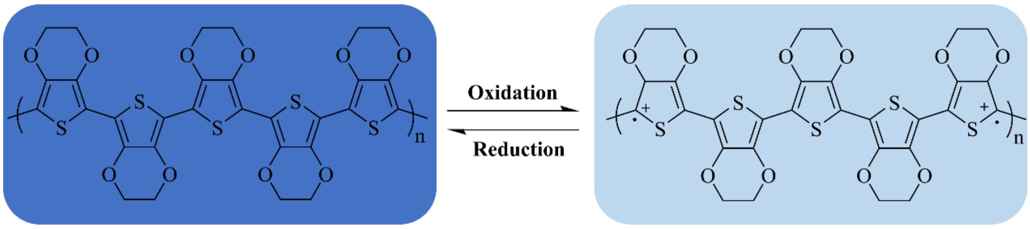

Figure S1. Schematic of the change in the optical color and transmittance of PEDOT films relying on the change in the oxidation–reduction state in line with the variations in the degree of doping.

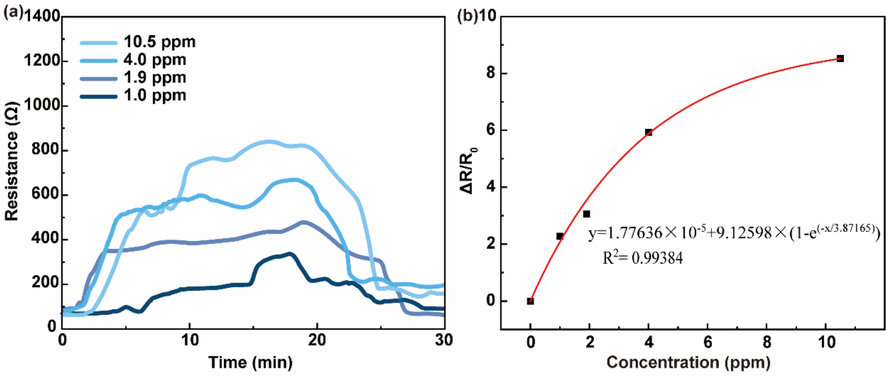

Figure S2. (a) Resistance–time curves and (b) resistance response–HPV concentration fitting curve of PEDOT:PSS-ATO films.

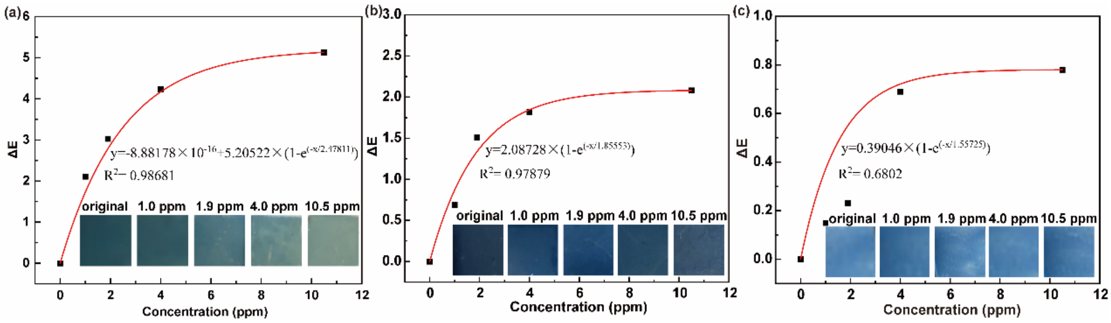

Figure S3. (a) Colorimetric response–HPV concentration fitting curve of PEDOT:PSS films. Inset: photos of the PEDOT:PSS film before and after testing different concentrations of HPV. Colorimetric response–HPV concentration fitting curve of (b) PEDOT:PSS/PEDOT film and (c) PEDOT film. Inset: photos of the PEDOT:PSS/PEDOT and PEDOT films before and after testing different concentrations of HPV.

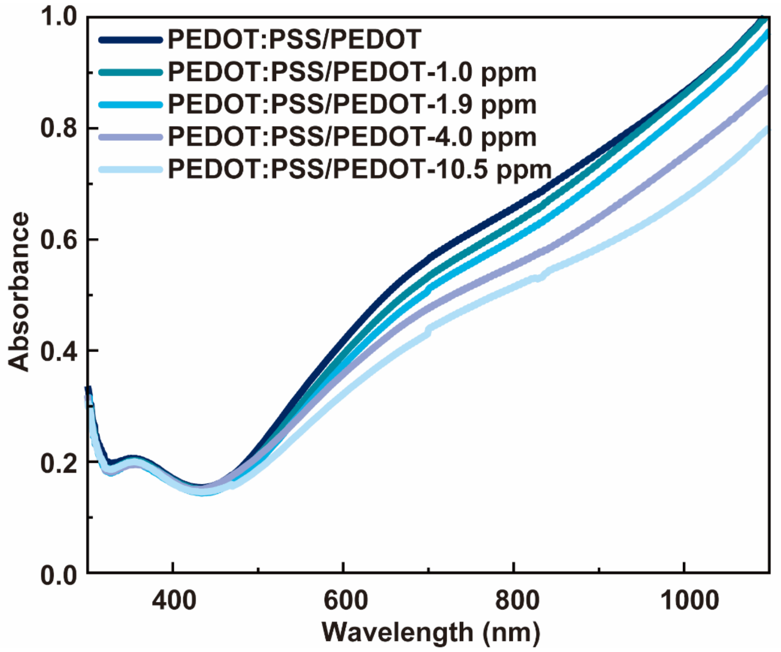

Figure S4. UV–vis absorption spectrum of the PEDOT:PSS/PEDOT film before and after the detection of different concentrations of HPV.
